# Unveiling the menace: a thorough review of potential pandemic fungal disease

**DOI:** 10.3389/ffunb.2024.1338726

**Published:** 2024-04-22

**Authors:** Mahdi Jafarlou

**Affiliations:** Department of Human Anatomy, Faculty of Medicine and Health Sciences, Universiti Putra Malaysia, Serdang, Selangor, Malaysia

**Keywords:** fungal diseases, global health threat, outbreaks, morbidity and mortality, pandemic, climate change, globalization

## Abstract

Fungal diseases have emerged as a significant global health threat, with the potential to cause widespread outbreaks and significant morbidity and mortality. Anticipating future pandemic fungal diseases is essential for effective preparedness and response strategies. This comprehensive literature review aims to provide a comprehensive analysis of the existing research on this topic. Through an extensive examination of scholarly articles, this review identifies potential fungal pathogens that have the potential to become pandemics in the future. It explores the factors contributing to the emergence and spread of these fungal diseases, including climate change, globalization, and antimicrobial resistance. The review also discusses the challenges in diagnosing and treating these diseases, including limited access to diagnostic tools and antifungal therapies. Furthermore, it examines the strategies and interventions that can be employed to mitigate the impact of future pandemic fungal diseases, such as improved surveillance systems, public health education, and research advancements. The findings of this literature review contribute to our understanding of the potential risks posed by fungal diseases and provide valuable insights for public health professionals and policymakers in effectively preparing for and responding to future pandemic outbreaks. Overall, this review emphasizes the importance of proactive measures and collaborative efforts to anticipate and mitigate the impact of future pandemic fungal diseases.

## 1 Introduction

The emergence and spread of infectious diseases have posed significant threats for devastating consequences on human populations, agriculture, and ecosystems. While viral and bacterial infections have traditionally received more attention in the context of pandemics, recent outbreaks, such as the emergence of COVID-19, have highlighted the need to also anticipate and prepare for future pandemic fungal diseases (Baker et al., 2022).

Fungi are a highly diverse group of organisms capable of causing various diseases in humans, plants, and animals. They can cause a pandemic due to their adaptability, spread to new regions, vulnerable populations, immunocompromised individuals, misuse of antifungal drugs, drug-resistant strains, climate change, and deforestation. Understanding this link is crucial for future preparedness. Some of important fungal infections in humans, include Candidiasis, which is caused by *Candida* species and commonly affects the skin, nails, and mucous membranes. Aspergillosis is another fungal infection in humans caused by Aspergillus species, primarily affecting the respiratory system. Cryptococcosis is caused by *Cryptococcus neoformans* and *Cryptococcus gattii*, and it can lead to severe lung and central nervous system infections. Histoplasmosis is caused by the fungus *Histoplasma capsulatum* and primarily affects the lungs, but it can also spread to other organs. Pneumocystis pneumonia is a serious fungal infection caused by the fungus *Pneumocystis jirovecii*. It primarily affects individuals with weakened immune systems, such as those with HIV/AIDS. It can cause severe respiratory symptoms, including coughing, shortness of breath, and fever (Rajendra Santosh et al., 2021), and Mucoromycosis, also known as zygomycosis, is a rare but serious fungal infection caused by fungi of the order Mucorales. It primarily affects individuals with weakened immune systems, such as those with uncontrolled diabetes, hematological malignancies, organ transplantation, or those undergoing treatment with immunosuppressive drug (Smith and Lee, 2022).

In plants, fungal diseases are widespread and can cause significant damage to crops, such as; Fusarium Wilt, caused by various Fusarium species, affects a wide range of plants, including tomatoes, bananas, and cotton, leading to wilting and death of the infected plants (Rokas, 2022).

In animals, fungal infections are also prevalent. Ringworm, caused by dermatophyte fungi, affects the skin, hair, and nails of various animals, including cats, dogs, and livestock (Seyedmousavi et al., 2018). Chytridiomycosis is a fungal disease that affects amphibians, caused by the chytrid fungus *Batrachochytrium dendrobatids*. It has been responsible for the decline and extinction of numerous amphibian species worldwide (Fisher et al., 2012).

Understanding the factors that contribute to the emergence and spread of fungal diseases is crucial for effective preparedness and prevention strategies. Environmental changes, such as climate change, deforestation, increased population density and urbanization as well as global travel and migration can create favorable conditions for fungal growth and transmission. For instance, deforestation can lead to increased contact between humans and wildlife, increasing the risk of zoonotic fungal infections. Climate change, on the other hand, can alter the geographical distribution of fungal pathogens, enabling them to establish themselves in new regions (Nnadi and Carter, 2021).

In addition to environmental factors, human activities also play a significant role in the emergence of fungal pandemics. The widespread use of broad-spectrum antibiotics and immunosuppressive drugs, invasive medical procedures and devices has led to an increase in opportunistic fungal infections, particularly in immunocompromised individuals. Furthermore, the global movement of people and goods has facilitated the rapid spread of fungal pathogens across borders, making containment and control challenging (Tarrant et al., 2021; Rokas, 2022).

Recent outbreaks, like the COVID-19 pandemic, have highlighted the importance of preparing for future pandemics, including those caused by fungal diseases. The global impact of COVID-19 has demonstrated how infectious diseases can rapidly spread and affect human, animal, and environmental health. Fungal pandemics, such as the emergence of multidrug-resistant *Candida auris*, can lead to significant morbidity and mortality. Strengthening surveillance, investing in research for antifungal drugs, improving diagnostics, and implementing robust infection prevention measures are key to enhancing global preparedness and response to fungal diseases. By integrating lessons from recent outbreaks into pandemic preparedness plans, we can better anticipate and mitigate the impact of future fungal pandemics (Haldane et al., 2021; Baker et al., 2022).

Anticipating future pandemic fungal diseases requires a multidisciplinary approach that integrates epidemiology, genomics, ecology, and public health. Surveillance systems need to be strengthened to detect and monitor emerging fungal pathogens and their potential for human-to-human transmission (Smith et al., 2023). Advances in genomics and molecular diagnostics can aid in the identification and characterization of novel fungal pathogens, facilitating the development of targeted therapies and vaccines (Tsalik et al., 2018; Malone et al., 2020; Okeke and Ihekweazu, 2021).

Furthermore, collaboration among epidemiology, genomics, ecology, and public health is crucial to address the challenges of fungal pandemics. Epidemiologists study the patterns and transmission of fungal diseases, while genomics provides insights into the genetic makeup and evolution of fungal pathogens. Ecologists examine the ecological factors influencing disease emergence, and public health professionals develop strategies for prevention and control. By sharing data and expertise, these fields can enhance understanding, develop evidence-based interventions, and safeguard public health (Gulis and Fujino, 2015; Gardy and Loman, 2018; Khoury et al., 2020).

## 2 Potential pandemic fungal pathogens

Several fungal species have demonstrated the potential to cause severe outbreaks and pose substantial threats to public health:

### 2.1 Candida auris

*Candida auris* is an emerging multidrug-resistant fungal pathogen that has gained attention due to its ability to cause severe infections and its capacity to persist in healthcare environments (Du et al., 2020). Its pathogenicity is attributed to several factors, including environmental contamination, rapid spreading, intrinsic and acquired resistance to antifungal drugs and disinfectants, its ability to adhere to and invade host tissues, and its ability to form biofilms (Caceres et al., 2019; Du et al., 2020; Horton and Nett, 2020; Ahmad and Alfouzan, 2021; Sharma and Goel, 2022).

*C. auris* has been found to persist in the environment, particularly in healthcare settings. Studies have shown that the fungus can survive on various surfaces, such as bed rails, furniture, and medical equipment, leading to environmental contamination and increasing the risk of transmission (Ahmad and Alfouzan, 2021). Also, it has been associated with outbreaks in healthcare facilities and has the potential to spread rapidly within these settings. Person-to-person transmission has been observed, and the fungus can also spread to the general population through healthcare workers or contaminated equipment (Jeffery-Smith et al., 2018; Du et al., 2020; Ahmad and Asadzadeh, 2023). It has been found to have intrinsic resistance to multiple classes of antifungal drugs, including azoles, echinocandins, and polyenes. This intrinsic resistance is thought to be due to genetic changes in the target enzymes or membrane components that are involved in drug susceptibility (Chowdhary et al., 2020). One of the key factors that contributes to the pathogenicity of *C. auris* is its ability to adhere to host tissues and form biofilms. Biofilms are complex communities of microorganisms encased in a self-produced extracellular matrix, which protects the organisms from host immune responses and antimicrobial treatments. *C. auris* has been shown to form robust biofilms on various surfaces, including medical devices and human tissues, allowing it to establish persistent infections (Jeffery-Smith et al., 2018; Caceres et al., 2019).

Furthermore, *C. auris* has been associated with high mortality rates in infected individuals, particularly those with underlying health conditions or compromised immune systems. The ability of *C. auris* to cause invasive infections, such as bloodstream infections, has been linked to its ability to disseminate within the host and evade host immune responses (Du et al., 2020; Ahmad and Alfouzan, 2021).

It is important to note that the exact mechanisms of *C. auris* pathogenicity are still being investigated, and there is ongoing research to better understand its virulence factors and the host immune responses to this pathogen.

### 2.2 Aspergillus fumigatus

*Aspergillus fumigatus* is a ubiquitous environmental fungus which is considered the most clinically significant species within the genus Aspergillus. It primarily affects the respiratory system and can spread to other organs, leading to high mortality rates. Its pathogenicity is attributed to several factors, including its ability to produce a variety of virulence factors and its ability to adapt and survive in different environments (Jafarlou et al., 2008; van de Veerdonk et al., 2017).

One of the key virulence factors of *A. fumigatus* is its production of secondary metabolites, such as gliotoxin. Gliotoxin is a potent immunosuppressive and cytotoxic compound that can inhibit the function of immune cells, such as neutrophils, macrophages, and dendritic cells, thereby allowing the fungus to evade the host immune response (Knowles et al., 2020).

Another important virulence factor is the ability of *A. fumigatus* to form biofilms. Biofilms are complex microbial communities that adhere to surfaces and are encased in a self-produced extracellular matrix. *A. fumigatus* biofilms have been shown to enhance the resistance of the fungus to antifungal drugs and to host immune defenses, allowing for persistent infection (Liu et al., 2022).

In addition, *A. fumigatus* produces enzymes, such as proteases and elastases, which can degrade host tissues and contribute to tissue invasion and damage (Ghazaei, 2017).

The ability of *A. fumigatus* to grow at physiological temperature (37°C) is also a critical factor in its pathogenicity. Unlike many other Aspergillus species that primarily cause infections in plants and animals, *A. fumigatus* has adapted to survive and grow at human body temperature, allowing it to colonize and infect human hosts (Mousavi et al., 2016).

Furthermore, the presence of asexual spores called conidia is another important aspect of *A. fumigatus* pathogenicity. The small size and hydrophobic nature of the conidia allow them to be easily inhaled and reach the lower respiratory tract. Once in the lungs, conidia can germinate and establish infection (Blatzer and Latgé, 2021).

It is worth noting that the pathogenesis of *A. fumigatus* infections is complex and involves interactions between the fungus and the host immune system and has shown increasing resistance to antifungal drugs, making treatment options limited. Climate change and environmental factors may contribute to the expansion of *A. fumigatus* habitats, increasing the risk of human exposure and potential for a pandemic (Verweij et al., 2016; van de Veerdonk et al., 2017).

### 2.3 Cryptococcus neoformans

*Cryptococcus neoformans* is a globally distributed fungal pathogen that primarily affects individuals with weakened immune systems, such as those with HIV/AIDS or undergoing immunosuppressive therapy (Rathore et al., 2022; Zhao et al., 2023). Its pathogenicity is attributed to several factors, including its ability to evade immune responses, its production of virulence factors, and its capacity to form a protective polysaccharide capsule. One of the key factors contributing to the pathogenicity of *C. neoformans* is its ability to evade immune responses. The polysaccharide capsule surrounding the fungus plays a crucial role in evading phagocytosis by host immune cells. The capsule inhibits recognition by host immune cells and interferes with the activation of immune response pathways, allowing the pathogen to survive and proliferate within the host (Alanio et al., 2015; Zaragoza, 2019; Rathore et al., 2022).

*C. neoformans* also produces various virulence factors that contribute to its pathogenicity. One such factor is melanin, which is produced by the fungus and provides protection against oxidative stress and phagocytosis. Melanin also inhibits the production of pro-inflammatory cytokines, further aiding the fungus in evading immune responses (Guerrero et al., 2006).

Furthermore, *C. neoformans* can undergo a process called phenotypic switching, where it can switch between different phenotypes, such as smooth and rough colonies. The smooth phenotype, associated with a thicker capsule, is more virulent and resistant to phagocytosis compared to the rough phenotype (Fries et al., 2002; Guerrero et al., 2006; Jain and Fries, 2008).

Another important aspect of *C. neoformans* pathogenicity is its ability to cause meningoencephalitis, a severe infection of the brain and meninges. The fungus can cross the blood–brain barrier and invade the central nervous system, leading to inflammation and neurological symptoms. The production of certain enzymes and factors, such as phospholipase and urease, by *C. neoformans* may contribute to its ability to invade and cause damage in the central nervous system (Bloom et al., 2019; Chen et al., 2022).

The emergence of multidrug-resistant strains and the potential for human-to-human transmission highlight the need for vigilance in monitoring and controlling this pathogen (Zhao et al., 2023).

### 2.4 Histoplasma capsulatum

*Histoplasma capsulatum* is a dimorphic fungus found in soil contaminated with bird or bat droppings. It causes histoplasmosis, a respiratory infection that can progress to disseminated disease in immunocompromised individuals. Its pathogenicity is attributed to its ability to survive within host macrophages, evade immune responses, and establish chronic infections in the host (Mittal et al., 2019; Nemade and Shinde, 2021).

One of the key factors contributing to the pathogenicity of *H. capsulatum* is its ability to survive and replicate within host macrophages. Upon inhalation of fungal spores, *H. capsulatum* is phagocytosed by alveolar macrophages. However, instead of being destroyed, the fungus can survive and replicate within these phagocytes. It can use several mechanisms to manipulate the host cell machinery and create an intracellular niche favorable for its growth (Deepe, 2000; Garfoot and Rappleye, 2016).

*H. capsulatum* also possesses the ability to evade immune responses. The fungus can modulate the host immune system by interfering with the activation of immune cells and suppressing the production of pro-inflammatory cytokines. This allows the fungus to establish a chronic infection and avoid clearance by the immune system (Mittal et al., 2019; Valdez et al., 2022).

Furthermore, *H. capsulatum* produces various virulence factors that contribute to its pathogenicity. One of the major virulence factors is a heat shock protein called Hsp60, which is involved in the survival and replication of the fungus within host cells. Hsp60 can also modulate host immune responses and contribute to the persistence of infection (Mihu and Nosanchuk, 2012; Mittal et al., 2019; Valdez et al., 2022).

The ability of *H. capsulatum* to form yeast cells is another crucial aspect of its pathogenicity. The transition from the mycelial form to the yeast form is essential for the establishment of infection. The yeast form of *H. capsulatum* is better adapted to survive within host tissues and evade immune recognition compared to the mycelial form (Mittal et al., 2019).

Histoplasmosis is endemic in certain regions, but its potential for dissemination through travel and migration raises concerns for a pandemic. Climate change and urbanization may also contribute to the expansion of *H. capsulatum* habitats, increasing the risk of exposure (Mittal et al., 2019; Linder and Kauffman, 2019).

### 2.5 Pneumocystis jirovecii

*Pneumocystis jirovecii* is an opportunistic fungal pathogen that primarily affects immunocompromised individuals, particularly those with weakened immune systems such as HIV/AIDS patients. Understanding its pathogenicity is crucial for the diagnosis, treatment, and prevention of Pneumocystis pneumonia (Gingerich et al., 2021; Weyant et al., 2021).

The exact mechanisms of *P. jirovecii* pathogenicity are not fully understood, but several factors contribute to its ability to cause disease. One of the main factors is its unique cell wall composition, which allows it to evade host immune responses. The cell wall of *P. jirovecii* contains complex polysaccharides that are thought to play a role in immune evasion and modulation of host immune responses (Apostolopoulou and Fishman, 2022). *P. jirovecii* also possesses surface proteins that may contribute to its pathogenicity. These proteins, such as kexin-like protein and major surface glycoprotein, are involved in adhesion to host cells and host immune evasion (Ma et al., 2016; Schmid-Siegert et al., 2021). Furthermore, *P. jirovecii* is believed to have a high degree of genetic diversity, which may contribute to its ability to evade host immune responses and adapt to different hosts. Genetic variations in *P. jirovecii* strains have been associated with differences in disease severity and clinical outcomes (White et al., 2019; Bateman et al., 2020).

The pathogenicity of *P. jirovecii* is also influenced by the host immune status. In immunocompromised individuals, such as those with HIV/AIDS, the weakened immune system allows for uncontrolled growth and dissemination of the fungus, leading to Pneumocystis pneumonia. The specific immune responses involved in controlling *P. jirovecii* infection are not fully understood, but both cell-mediated and humoral immune responses are thought to play a role (Gülbudak et al., 2020; Charpentier et al., 2021). *P. jirovecii* is transmitted through respiratory droplets and has the potential for human-to-human transmission (Vera and Rueda, 2021). The emergence of drug-resistant strains and the increasing population of immunocompromised individuals worldwide contribute to the potential for a pandemic (Phipps et al., 2011; Vera and Rueda, 2021).

### 2.6 Mucormycetes

Mucormycetes, also known as zygomycetes, are filamentous fungi that can cause mucormycosis or zygomycosis in humans, particularly in individuals with compromised immune systems which are associated with high morbidity and mortality rates (Smith and Lee, 2022). Mucormycetes can enter the human body through inhalation, ingestion, or direct contact with contaminated materials. Once inside the host, these fungi have the ability to invade blood vessels, leading to tissue necrosis and the formation of characteristic fungal hyphae. The invasion of blood vessels is a key factor in their pathogenicity, as it allows for rapid spread and extensive tissue damage (Sharma and Goel, 2022).

The pathogenicity of mucormycetes is attributed to their mechanisms of iron acquisition and secretion of various enzymes and toxins. These fungi produce siderophores, which are iron-scavenging molecules that enable them to survive and proliferate in iron-limited environments, such as the human body. Additionally, they secrete enzymes, like proteases, that degrade host tissues and facilitate invasion. Furthermore, mucormycetes release mycotoxins, such as gliotoxin, which can suppress the immune response and promote fungal survival (Skiada et al., 2020; Sharma and Goel, 2022).

Early diagnosis of mucormycosis is often challenging due to nonspecific symptoms that can mimic other infections. Consequently, mucormycosis is frequently diagnosed at advanced stages when the infection has already spread. Moreover, mucormycetes are inherently resistant to many antifungal drugs, including azoles, which are commonly used for fungal infections. This limited availability of effective antifungal agents complicates treatment options for mucormycosis (Skiada et al., 2020; Sharma and Goel, 2022; Smith and Lee, 2022).

In recent times, there has been a concerning association between mucormycosis and COVID-19, leading to the term “COVID-19 associated mucormycosis” or “black fungus.” COVID-19 patients with compromised immune systems, especially those receiving corticosteroid therapy, have been reported to be at a higher risk of developing mucormycosis. The combination of COVID-19 infection and mucormycosis poses significant challenges in diagnosis and treatment, further emphasizing the need for early detection and a multidisciplinary approach to manage these cases effectively (Singh et al., 2021; Hoenigl et al., 2022).

## 3 Virulence factors and therapeutic potential of fungal pathogens

Fungal pathogens have evolved various virulence factors and mechanisms that contribute to their pathogenicity. Understanding these factors is crucial for developing targeted therapeutic strategies. Some of well-known and important virulence factors exhibited by fungal pathogens and their potential as treatment targets are:

### 3.1 Adhesion and invasion

Fungal pathogens possess adhesins, such as *Candida albicans* Als proteins or *Aspergillus fumigatus* hydrophobins, which facilitate attachment to host tissues (Naglik et al., 2003; Aimanianda and Latgé, 2010). These adhesins promote invasion and colonization of host cells. Targeting these adhesion molecules may disrupt fungal-host interactions and prevent infection.

### 3.2 Secreted enzymes

Fungal pathogens secrete various enzymes that enable tissue invasion and nutrient acquisition. For example, *Candida* species produce secreted aspartyl proteinases (Saps) that degrade host proteins, impairing immune defenses (Naglik et al., 2003). Inhibiting these enzymes could potentially hinder fungal invasion and reduce tissue damage.

### 3.3 Toxin production

Certain fungal species produce toxins that contribute to their virulence. *Aspergillus fumigatus* releases gliotoxin, which inhibits immune cell function and promotes fungal survival (Spikes et al., 2008). Targeting toxin production pathways may offer therapeutic opportunities to weaken fungal virulence and enhance host immune responses.

### 3.4 Biofilm formation

Fungal pathogens, such as *Candida* species, can form biofilms on medical devices, leading to persistent infections. Biofilms provide protection against host immune responses and antimicrobial agents. Disrupting biofilm formation mechanisms, such as targeting key regulatory genes or inhibiting extracellular matrix production, could enhance treatment efficacy (Malinovská et al., 2023).

### 3.5 Antifungal drug resistance

Fungal pathogens can acquire resistance to commonly used antifungal drugs, posing a significant challenge for treatment. The emergence of multidrug-resistant fungal pathogens is a significant concern in healthcare settings (Fisher et al., 2012). These resistant strains demonstrate resistance to multiple classes of antifungal drugs, making them difficult to treat and control. The spread of such resistant strains within healthcare facilities poses a serious threat to vulnerable patient populations (Vallabhaneni et al., 2015; Fisher et al., 2022).

One primary mechanism of antifungal resistance is the acquisition of genetic mutations or the transfer of resistance genes from other organisms. These mutations or genes can confer reduced susceptibility or complete resistance to antifungal drugs. Fungal pathogens can also employ efflux pumps to actively remove antifungal drugs from within their cells, reducing their effectiveness (Lee et al., 2023).

### 3.6 Global burden of fungal infections

Fungal infections pose a significant global burden, and several factors contribute to their impact on public health. One key factor is the difficulty in diagnosing fungal infections accurately and promptly. Many fungal infections present with nonspecific symptoms that overlap with other conditions, leading to delays in diagnosis and treatment initiation. This delay can result in the progression of the infection, increased morbidity, and mortality rates (Bongomin et al., 2017).

Another crucial factor is the absence of effective vaccines for most fungal infections. Unlike bacterial or viral infections, vaccines for fungal pathogens are limited (Brown et al., 2012). Vaccines play a vital role in preventing infectious diseases by stimulating the immune system to recognize and mount a defense against specific pathogens. The lack of effective vaccines leaves individuals susceptible to fungal infections and limits preventive measures (Brown et al., 2012; Tripathi, 2023).

Transmissibility is another important factor contributing to the global burden of fungal infections. Some fungal pathogens, such as *Candida auris*, have demonstrated the ability to spread rapidly and cause outbreaks in healthcare settings (Vallabhaneni et al., 2015). These multidrug-resistant strains can colonize surfaces and persist in the environment, making them difficult to eradicate. This transmissibility within healthcare facilities increases the risk of fungal infections and contributes to the global burden (Vallabhaneni et al., 2015; Parmanik et al., 2022).

The limited treatment options for fungal infections also contribute to their global impact. Fungal resistance to antifungal drugs has become a significant concern (Wiederhold, 2017). Fungal pathogens can acquire genetic mutations or resistance genes, reducing their susceptibility or conferring complete resistance to antifungal agents (Lee et al., 2023). This limits the available treatment options and increases the risk of treatment failure. The emergence of multidrug-resistant fungal strains further exacerbates this problem (Lee et al., 2021, 2023).

Furthermore, immunosuppressed populations, such as individuals with HIV/AIDS, organ transplant recipients, or patients undergoing chemotherapy, are particularly vulnerable to fungal infections. Their weakened immune response makes it harder to control and eliminate fungal pathogens, leading to more severe and recurrent infections (Pappas et al., 2016).

Addressing the global burden of fungal infections requires a multidimensional approach. This includes investing in research and development for new antifungal drugs with novel mechanisms of action, improving diagnostic methods for accurate and rapid identification of fungal infections, developing effective vaccines, implementing infection prevention and control measures in healthcare settings, and raising awareness among healthcare professionals and the general public about the importance of early detection and appropriate management of fungal infections.

## 4 Contributing factors to fungal pandemics

Several factors contribute to the emergence and spread of fungal diseases, potentially leading to a pandemic. Some specific examples and statistics which highlight the impact of fungal habitats and human exposure to pathogenic fungi are:

### 4.1 Indoor environments

Indoor spaces can serve as habitats for various fungi, including pathogenic species. For example, a study conducted in the United States found that approximately 45% of homes had visible mold growth (Mendell et al., 2011). Exposure to indoor molds has been associated with respiratory symptoms, allergies, and asthma exacerbations.

### 4.2 Healthcare-associated infections

Fungal infections acquired in healthcare settings, known as healthcare-associated infections (HAIs), are a significant concern. According to the Centers for Disease Control and Prevention (CDC), *Candida* species account for the majority of fungal HAIs in the United States (Haque et al., 2020). *Candida* infections can range from mild oral thrush to life-threatening bloodstream infections, with an estimated mortality rate of 40–60% for *Candida* bloodstream infections.

### 4.3 Community-acquired fungal infections

Fungal infections can also be acquired outside of healthcare settings. For instance, coccidioidomycosis, also known as Valley fever, is a fungal infection caused by the soil-dwelling fungus Coccidioides. It is endemic in certain regions of the United States, primarily in the southwestern states. In 2019, there were over 16,000 reported cases of coccidioidomycosis in the United States (Williams and Chiller, 2022).

### 4.4 Agricultural and occupational exposure

Individuals working in certain occupations, such as agriculture or construction, may be at an increased risk of exposure to pathogenic fungi. Farmworkers, for example, can be exposed to fungi present in soil, crops, and organic matter. Occupational exposure to fungi has been associated with respiratory diseases, such as farmer’s lung and humidifier lung (Rylander, 1986).

### 4.5 Immunocompromised individuals

People with weakened immune systems, such as those living with HIV/AIDS, undergoing chemotherapy, or receiving organ transplants, are particularly vulnerable to fungal infections. Invasive fungal infections, such as invasive aspergillosis or cryptococcosis, can be life-threatening in these individuals. According to the Global Action Fund for Fungal Infections (GAFFI), an estimated 1.6 million deaths occur annually due to fungal diseases worldwide, with the majority of these deaths occurring in immunocompromised individuals (Bongomin et al., 2017).

These examples and statistics demonstrate the diverse range of fungal habitats and the potential for human exposure to pathogenic fungi. It is essential to understand and address these exposures to mitigate the impact of fungal infections on human health. Public health measures, such as improving ventilation in indoor spaces, implementing infection control practices in healthcare settings, and raising awareness among high-risk populations, can help reduce the burden of fungal diseases.

## 5 Prevention and control strategies

To effectively prevent and control future pandemic fungal diseases, a multifaceted approach is required. Enhanced surveillance systems are critical for early detection and monitoring of emerging fungal pathogens. These systems should include rapid diagnostic tools and techniques to accurately identify fungal infections, enabling timely treatment and containment. Molecular techniques, such as polymerase chain reaction (PCR) and next-generation sequencing, have shown promise in improving the speed and accuracy of fungal diagnosis (McCarthy et al., 2017).

Public awareness campaigns targeting healthcare providers and the general population are crucial in preventing the spread of fungal diseases. Education should focus on recognizing risk factors, early signs and symptoms, and appropriate prevention strategies. In healthcare settings, infection control practices, including proper hand hygiene, appropriate use of personal protective equipment, and maintenance of environmental cleanliness, are essential for preventing nosocomial transmission of fungal infections (Haque et al., 2020).

Research and development of new antifungal drugs and therapies are necessary to combat emerging multidrug-resistant fungal pathogens. The current arsenal of antifungal agents is limited, and resistance is a growing concern. Antifungal stewardship programs should be implemented to promote rational use of antifungal agents, prevent the emergence of resistance, and optimize patient outcomes (Fisher et al., 2022). Additionally, efforts should be directed towards developing vaccines against fungal pathogens to provide a proactive approach to prevention (Wall and Lopez-Ribot, 2020).

## 6 Conclusion

While viral and bacterial pandemics have dominated recent global health concerns, the potential threat of fungal pandemics should not be overlooked. *Candida auris* and *Aspergillus fumigatus* are examples of fungal pathogens that have demonstrated the ability to cause severe outbreaks and spread rapidly. Contributing factors such as climate change, environmental disturbances, and medical interventions increase the risk of fungal infections.

Implementing strategies such as enhanced surveillance, improved diagnostics, antifungal stewardship, and infection control practices will be crucial in mitigating the impact of future pandemic fungal diseases. However, to effectively address this emerging global health threat and prepare our defenses against all types of infectious pathogens, we need to take specific steps.

Promoting further research is paramount. By advocating for increased research efforts on fungal pandemics, we can enhance our understanding of these diseases and develop better prevention and treatment strategies. This research should focus on exploring the mechanisms of fungal pathogenesis, identifying potential drug targets, and developing new antifungal agents. Moreover, research should also investigate the impact of climate change and environmental factors on the emergence and spread of fungal infections.

Advocating for policy changes is another crucial step. We need policies that support funding for research on fungal pandemics, as well as policies that prioritize the development and implementation of infection control measures. Governments and international organizations should allocate resources to strengthen healthcare infrastructure, enhance laboratory capacity, and promote education and training programs for healthcare professionals.

Supporting global collaborations is vital in effectively combating fungal pandemics. Fungal infections do not recognize borders, and international cooperation is essential in sharing knowledge, expertise, and resources. Collaborative efforts can include establishing global surveillance networks to monitor the spread of fungal pathogens, sharing best practices in infection control, and coordinating responses during outbreaks. By working together, we can leverage our collective strengths and respond more effectively to fungal pandemics.

Emphasizing the need for preparedness is crucial. We must invest in early detection methods, such as rapid diagnostic tests, to quickly identify and isolate infected individuals. Building global surveillance networks will enable us to monitor the emergence and spread of fungal pathogens in real time. Additionally, enhancing medical infrastructure, including the availability of antifungal medications and the training of healthcare professionals, is essential to effectively respond to future outbreaks.

It is important to highlight the global nature of the issue and emphasize the importance of international cooperation, knowledge-sharing, and resource allocation. Fungal pandemics affect populations worldwide, and no single country can tackle this challenge alone. By working together, we can share experiences, learn from each other’s successes and failures, and allocate resources where they are most needed. This global collaboration is crucial in effectively combating fungal pandemics and protecting global health. By taking these steps, we can enhance our understanding of fungal infections, develop effective strategies to prevent and treat them, and ensure a coordinated global response. Together, we can mitigate the impact of fungal pandemics and protect the health and well-being of people around the world.

## Author contributions

MJ: Conceptualization, Data curation, Formal Analysis, Investigation, Resources, Supervision, Writing – original draft, Writing – review & editing.
